# Reply to: Methodological concerns regarding stepwise regression and construct overlap in caregiver burden research

**DOI:** 10.1590/1980-5764-DN-2026-0475R

**Published:** 2026-07-20

**Authors:** Andressa Hermes-Pereira, Mariana Goulart, Renata Kochhann, Artur Francisco Schumacher-Schuh

**Affiliations:** 1 Universidade Federal do Rio Grande do Sul, Department of Pharmacology, Porto Alegre RS, Brazil.; 2 Pontifícia Universidade Católica do Rio Grande do Sul, Postgraduate Program in Psychology, Porto Alegre RS, Brazil.; 3 Hospital Moinhos de Vento, Memory Center, Porto Alegre RS, Brazil.; 4 Hospital de Clínicas de Porto Alegre, Neurology Service, Porto Alegre RS, Brazil.

## INTRODUCTION

Dear Editor,

We thank Dr. de Araujo for his knowledgeable comments on our study. The main concern was the use of stepwise regression analysis to select predictors of burden. We agree with the limitations regarding this analysis. However, it continues to be used, and the benefits of stepwise regression analysis are primarily its ability to automate variable selection in multivariable models, especially when there is limited knowledge of which variables might be predictive^1,2^.

To assess each variable in relation to caregiver burden, we include linear simple regression for each independent variable as follows. Furthermore, we need to include a correction in the method because there was a mistake in the writing: only patient functionality was not statistically significantly correlated with caregiver burden. The other variables (anxious symptoms and the severity of neuropsychiatric symptoms) were excluded by the stepwise analysis.

Simple linear regression analysis was conducted for each variable (depressive symptoms, caregiver distress, quality of life, anxious symptoms, severity of neuropsychiatric symptoms, and patient functionality) to assess the extent to which each could independently predict caregiver burden. Depression symptoms explained approximately 36% of the variance in caregiver burden (F(1,126)=71.71, p<0.001). Similarly, caregiver distress explained approximately 34% of the variance in caregiver burden (F(1,126)=64.09, p<.001). Additionally, quality of life explained approximately 23% of the variance in caregiver burden (F(1,117)=35.20, p<.001). When evaluated in isolation, the symptoms of anxiety explained approximately 27% of the variance in caregiver burden (F(1,126)=47.57, p<.001). The severity of neuropsychiatric symptoms had the lowest contribution, explaining approximately 21% of the variance in caregiver burden (F(1,126)=33.36, p<.001). Finally, patient functionality was not significantly associated with caregiver burden (F(1,126)=0.79, p=.374).

Thus, it can be observed that the variables that best explained caregiver burden in isolation were depressive symptoms and caregiver distress. These variables, along with quality of life, have clinical relevance. Additionally, regarding the semantic overlap between BDI-II (Beck Depression Inventory – II) and ZBI (Zarit Burden Interview), it is important to point out that there is a possible overlap in only three items on a scale of 21.

In summary, we believe that our results support the hypothesis that depression symptoms, caregiver distress, and quality of life together can reasonably explain caregiver burden due to their clinical relevance, but perhaps not with the magnitude shown in the stepwise regression statistical analysis.

## Data Availability

The datasets generated and/or analyzed during the current study are available from the corresponding author upon reasonable request.
